# Can the intake of antiparasitic secondary metabolites explain the low prevalence of hemoparasites among wild Psittaciformes?

**DOI:** 10.1186/s13071-018-2940-3

**Published:** 2018-06-19

**Authors:** Juan F. Masello, Javier Martínez, Luciano Calderón, Michael Wink, Petra Quillfeldt, Virginia Sanz, Jörn Theuerkauf, Luis Ortiz-Catedral, Igor Berkunsky, Dianne Brunton, José A. Díaz-Luque, Mark E. Hauber, Valeria Ojeda, Antoine Barnaud, Laura Casalins, Bethany Jackson, Alfredo Mijares, Romel Rosales, Gláucia Seixas, Patricia Serafini, Adriana Silva-Iturriza, Elenise Sipinski, Rodrigo A. Vásquez, Peter Widmann, Indira Widmann, Santiago Merino

**Affiliations:** 1 Department of Animal Ecology and Systematics, Justus-Liebig Universität Gießen, Heinrich-Buff-Ring 26, D-35392 Gießen, Germany; 2 Departamento de Biomedicina y Biotecnologıía, Area Parasitologıía, Facultad de Farmacia, Universidad de Alcalá (UAH), NII Km 33.600, 28805 Alcalá de Henares, Madrid, Spain; 3 Institute of Pharmacy and Molecular Biotechnology, Heidelberg University, INF 364, 69120 Heidelberg, Germany; 4 Centro de Ecología, Instituto Venezolano de Investigaciones Científicas, Altos de Pipe, Venezuela; 5 Museum and Institute of Zoology, Polish Academy of Sciences, Wilcza 64, 00-679 Warsaw, Poland; 6 Institute of Natural and Mathematical Sciences, Massey University, Auckland, New Zealand; 7 Instituto Multidisciplinario sobre Ecosistemas y Desarrollo Sustentable, Universidad Nacional del Centro de la Provincia de Buenos Aires, Tandil, Argentina; 8 Fundación para la Investigación y la Conservación de los Loros en Bolivia (CLB), Avenida Francisco Mora, Santa Cruz de la Sierra, Bolivia; 9 Centro de Conservación de Loros Silvestres (CREA), Santa Cruz de la Sierra, Bolivia; 10 Department of Animal Biology, School of Integrative Biology, University of Illinois, Urbana-Champaign, IL 61801 USA; 11 ZoologyDepartment (CRUB-UNCo), INIBIOMA (Universidad Nacional del Comahue-CONICET), 8400 Bariloche, Argentina; 12 Province des Iles Loyauté, Direction du Développement Economique, BP 50 98820 Wé, Lifou New Caledonia; 13 Auckland Zoological Park, Motions Road, Western Springs, Auckland, 1022 New Zealand; 14 School of Veterinary and Life Sciences, Murdoch University, Perth, WA Australia; 15 Centro de Bioquímica y Biofísica, Instituto Venezolano de Investigaciones Científicas, Altos de Pipe, Venezuela; 16 Projeto Papagaio-verdadeiro, Fundação Neotropica do Brasil, Campo Grande, Brazil; 17 Base Multifuncional do CEMAVE em Florianópolis/SC, Estação Ecológica Carijós – ICMBio, Florianópolis, Brazil; 18 Projeto de Conservação do papagaio-de-cara-roxa, SPVS - Sociedade de Pesquisa em Vida Selvagem e Educação Ambiental, Curitiba, Brazil; 19 Institute of Ecology and Biodiversity, Departamento de Ciencias Ecológicas, Facultad de Ciencias Universidad de Chile, Santiago, Chile; 20 Katala Foundation, Inc., Puerto Princesa City, Palawan Philippines; 21 Departamento de Ecología Evolutiva, Museo Nacional de Ciencias Naturales, Consejo Superior de Investigaciones Científicas, 28006 Madrid, Spain

**Keywords:** Antiparasitic metabolites, Blood parasites, Cacatuidae, Haemoparasites, Herbivorous, Omnivorous, Plant secondary metabolites, Psittacidae, Self-medication

## Abstract

**Background:**

Parasites can exert selection pressure on their hosts through effects on survival, on reproductive success, on sexually selected ornament, with important ecological and evolutionary consequences, such as changes in population viability. Consequently, hemoparasites have become the focus of recent avian studies. Infection varies significantly among taxa. Various factors might explain the differences in infection among taxa, including habitat, climate, host density, the presence of vectors, life history and immune defence. Feeding behaviour can also be relevant both through increased exposure to vectors and consumption of secondary metabolites with preventative or therapeutic effects that can reduce parasite load. However, the latter has been little investigated. Psittaciformes (parrots and cockatoos) are a good model to investigate these topics, as they are known to use biological control against ectoparasites and to feed on toxic food. We investigated the presence of avian malaria parasites (*Plasmodium*), intracellular haemosporidians (*Haemoproteus*, *Leucocytozoon*), unicellular flagellate protozoans (*Trypanosoma*) and microfilariae in 19 Psittaciformes species from a range of habitats in the Indo-Malayan, Australasian and Neotropical regions. We gathered additional data on hemoparasites in wild Psittaciformes from the literature. We considered factors that may control the presence of hemoparasites in the Psittaciformes, compiling information on diet, habitat, and climate. Furthermore, we investigated the role of diet in providing antiparasitic secondary metabolites that could be used as self-medication to reduce parasite load.

**Results:**

We found hemoparasites in only two of 19 species sampled. Among them, all species that consume at least one food item known for its secondary metabolites with antimalarial, trypanocidal or general antiparasitic properties, were free from hemoparasites. In contrast, the infected parrots do not consume food items with antimalarial or even general antiparasitic properties. We found that the two infected species in this study consumed omnivorous diets. When we combined our data with data from studies previously investigating blood parasites in wild parrots, the positive relationship between omnivorous diets and hemoparasite infestation was confirmed. Individuals from open habitats were less infected than those from forests.

**Conclusions:**

The consumption of food items known for their secondary metabolites with antimalarial, trypanocidal or general antiparasitic properties, as well as the higher proportion of infected species among omnivorous parrots, could explain the low prevalence of hemoparasites reported in many vertebrates.

**Electronic supplementary material:**

The online version of this article (10.1186/s13071-018-2940-3) contains supplementary material, which is available to authorized users.

## Background

Parasites can exert ecological and evolutionary pressures on their hosts [1–4]. This pressure can affect the host in various ways, including decreased body condition, reproductive success, and survival, as well as physiological castration [5–8]. Ultimately, parasites affect the fitness of the host by promoting the evolution of behavioural, physiological or immunological anti-parasite defences [1, 9–11]. The co-evolutionary dynamics of host defence mechanisms and parasite counter-adaptations are influenced by many factors, including environmental conditions, genetic background, immune defence investment, life history traits, behaviour, host age and sex [12–16].

Due to their ecological and evolutionary importance, avian malaria parasites (*Plasmodium*) and related haemosporidians (*Haemoproteus* and *Leucocytozoon*), the unicellular parasitic flagellate protozoa *Trypanosoma*, and the early stage in the life-cycle of some parasitic nematodes known as microfilaria, have become the focus of a number of avian studies and has resulted in the establishment of an open access database dedicated to haemosporidians (MalAvi) [3, 16–23]. Avian haemosporidians are single-celled, intracellular parasites in which the fertilisation, formation of zygotes, and asexual sporogony take place in a blood-sucking dipteran vector while sexual gametogony and asexual merogony occur in the avian host [24]. *Plasmodium* haemosporidians are usually transmitted by dipteran vectors belonging to the Culicidae, while *Haemoproteus* are transmitted by dipterans of the Ceratopogonidae and Hippoboscidae, and *Leucocytozoon* through species belonging to the Simuliidae [24]. Avian *Trypanosoma* are transmitted by biting midges (Ceratopogonidae) [25], while in the case of microfilariae the common intermediate hosts are blood-sucking insects of the Simuliidae, Ceratopogonidae, Tabanidae and Culicidae [26]. Although all these blood parasites are widely distributed, they are restricted to the distribution of both their avian hosts and their vectors [24, 27, 28]. *Plasmodium* appears to be more cosmopolitan than the other related haemosporidians. *Haemoproteus* appears to be absent from some oceanic regions, while *Leucocytozoon* appears to be less abundant in the Neotropical and Australian regions [19, 24, 28, 29]. Blood parasites are distinguished by having at least one developmental stage in the host bloodstream (for detailed descriptions see [24]), and by eliciting chronic infections in wild birds [1–3, 24]. Yet, a relapse of the parasite infection usually takes place during the breeding season of the avian host, facilitating the infection of the vectors and the transfer of infection to the offspring [24]. Delayed reproduction, reduced clutch sizes, reduced parental working capacity while feeding nestlings, and increased predation risk, are some of the reported effects of blood parasites on the host, particularly during situations of stress that deteriorate the condition of an individual [1, 2, 7, 10, 26]. Although some blood parasites have been classified as non-pathogenic, they certainly remove resources from the host that could be otherwise be used for growth, maintenance or reproduction, reducing thus reproductive success and ultimately fitness [1].

The degree of blood parasite infection reported varies greatly among birds, including taxa with high prevalence (e.g. songbirds), but also taxa with low prevalence (e.g. some seabirds) or even an absence of parasites (e.g. storks, waders, nightjars, some seabirds, sandgrouse, parrots, swifts) [24, 30–44]. Various factors may explain this disparity. Songbirds have been more often sampled than some other bird groups that have not been recorded harbouring blood parasites [19, 24–44]. Secondly, microscopy may underestimate the prevalence in cases of very low infection, poor quality of the blood smears, or if the observers are not properly trained (e.g. [40, 41], and references therein). Various abiotic and biotic factors might also explain the large taxonomic variability of infection prevalence e.g. nesting habitat, nest characteristics, host density, temperature, elevation, topography, water availability and vector abundance [41, 45–47]. Several of these factors have been shown to influence the hemoparasite-host interactions by affecting parasite prevalence in the insect vectors and the probability of transmission to the avian host [20, 47–49]. Birds from certain habitats, such as tundra, arid, island and/or marine environments, have been reported as having a lower prevalence than birds from other habitats [19, 32, 43, 50–53]. Another possible reason for the variation in abundance of blood parasites includes the variable presence and density of the vectors [50, 54, 55], and the ability by the host immune system to resist or control infection [24]. Moreover, a blood parasite will reach its host only during a specific life-stage when the avian host has to be susceptible, the necessary vectors need to be present and competent, and the environmental conditions need to be appropriate for the transmission [47]. Food availability appears to play a key role by affecting the condition of the birds, which in turn can affect their susceptibility to parasites [56–58]. Feeding behaviour can likewise play an important role [12, 20]. A wide-ranging study on wild Neotropical birds found that species with omnivorous diets had a higher prevalence of *Plasmodium*, whereas insectivores had a higher prevalence of microfilariae [20]. Moreover, some animals consume food containing secondary metabolites with preventative or therapeutic effects that can be used as self-medication to reduce parasite load, against microbes or that can even serve as antioxidants [59–63]. These secondary metabolites often interact with proteins, biomembranes or nucleic acids of the parasites, disrupting their bioactivities and thus acting as effective antiparasitic medication [63]. Non-human primates, ruminants, wolves (*Canis lupus*), cougars (*Puma concolor*) and domestic dogs (*C. l. familiaris*) ingest plants with antiparasitic properties but with little or no nutritional value [59–61, 64–66], wood ants (*Formica paralugubris*) use resin to inhibit the growth of microorganisms [67], some passerines use lime rind against lice [68] and fresh plant material to repel parasites or mask the chemical cues that parasites use to find the host [69], while great bustards (*Otis tarda*) have been shown to consume blister beetles (Meloidae) that contain secondary metabolites with antimicrobial and pathogen-limiting activity [62]. Consequently, there is not a unique explanation for the complex variety of hemoparasite-host interactions, and comparative multivariate analyses suggest that phylogenetic, ecological, behavioural, climatic, and life-history traits determine the large variation observed in hemoparasite prevalence [3, 7, 12, 16, 19, 20, 43, 47, 48, 70–72]. Lastly, it is important mentioning that a reduction or the absence of parasite infections might influence the evolutionary trajectories of bird species. Identifying the underlying drivers of variation in pathogen prevalence has important ramifications in the fields of evolutionary ecology and disease ecology. Accordingly, the avoidance of infections may positively affect host traits, such as reproduction and survival, allowing species not subject to pathogen pressure to locally outcompete other species or to become successful invaders in introduced communities [73, 74].

The Psittaciformes (parrots and cockatoos) avian order is distributed from the tropics to sub-Antarctic regions, in a wide range of habitats extending from tundra to rainforest [75]. Psittaciformes are mostly cavity nesters, with only monk parakeets (*Myiopsitta monachus*) building twig nests and some *Agapornis* species building domed nests within cavities [75, 76]. Most species tend to be gregarious forming loose to very dense colonies [77–79]. The wide range of habitats used, the gregarious behaviour and the nesting characteristics of the Psittaciformes could favour contact with the vectors and hence the transmission of parasites. In fact, some haemosporidians like *Haemoproteus* (*Parahaemoproteus*) *handai*, *H.* (*P.*) *psittaci*, and *H.* (*P.*) *homohandai* have been originally described from captive Psittaciformes [24, 80, 81]. Moreover, blood parasites appear to be common among captive parrots particularly in zoos ([24, 80–82], most records in [83–89]). However, when considering only wild Psittaciformes, 66% of parrot populations studied so far reported an apparent absence of blood parasites (44 of 67 populations; Additional file 1: Table S1, and references therein). A plausible explanation for this difference is that the stress associated with captivity may increase the immunological susceptibility of individuals or reduce their capacity to avoid the vectors commonly present in zoos, thus increasing parasite load [78–92]. Another explanation is that the absence of hemoparasites could be related to a strong innate immunity in some Psittaciformes [42, 93]. Also, the monk parakeet and the red-fronted parakeet (*Cyanoramphus novaezelandiae*) have been shown to bring fresh green leaves to the nest, a behaviour that has been interpreted as a way to actively deter ectoparasites [94, 95] acting as blood parasite vectors. Many parrots in the wild feed on toxic fruits, seeds or flower buds [96–98], whereas in captivity they obtain food that does not contain toxins [99]. Thus, it could also be possible that parrots use secondary metabolites present in the diet as self-medication to reduce parasite load [61–69, 95]. Alternatively, differences in the diet, habitats or environmental factors like climate could also determine the large interspecific variation in blood parasite prevalence reported for Psittaciformes (Additional file 1: Table S1 and references therein). Until now, none of these potential explanations has been investigated in detail and information on blood parasite infection in wild Psittaciformes remains patchy, including mostly occasional data collected during general surveys (Additional file 1: Table S1 and references therein), and few individual species investigated in detail [100–102].

Given the wide range of habitats, climates and diets that Psittaciformes exploit, as well as their previously reported antiparasitic behaviour, this group of birds has a great potential as a model to investigate environmental and behavioural factors. This includes diet selection and self-medication, which may determine the interspecific variation in blood parasite prevalence in vertebrates. We therefore studied the presence of hemoparasites of the genera *Haemoproteus*, *Plasmodium*, *Leucocytozoon* and *Trypanosoma*, as well as microfilariae across populations of wild Psittaciformes. We sampled 19 Psittaciformes species from 25 localities covering an extensive range of habitats and climate types in the Indo-Malayan, Australasian and Neotropical zoogeographical regions. We considered extrinsic and intrinsic factors that may control the presence of hemoparasites in Psittaciformes, compiling information on food items consumed, habitats used and climate. We also investigated the potential role of the food consumed in providing antiparasitic secondary metabolites that could be used as self-medication to reduce parasite load. Furthermore, we searched the literature for wild parrots previously investigated for hemoparasites, and additionally compiled information on the corresponding diets, habitats and climates. We hypothesised that parrots consuming antiparasitic secondary metabolites have a lower prevalence of hemoparasites.

## Methods

### Own samples

Between 1999 and 2014, we obtained blood (*n* = 329), liver (*n* = 23), heart (*n* = 1) and kidney (*n* = 1) samples belonging to 19 Psittaciformes species from 25 localities. Maps showing the position of all localities sampled in this study are provided in Additional file 2: Figures S1–S5. Adults (*n* = 213), nestlings (*n* = 112) and juveniles (*n* = 4) were captured in their nests. The age of the adult parrots sampled was unknown. We obtained samples from nestlings during the pre-fledging (pf) period; this means the nesting period shortly before the young leave the nest and long after the minimal prepatent period reported for several blood parasites [103–105]. The prepatent period for blood parasites i.e. the period between infection and presence of infective forms in blood, varies between 5 and 14 days [24, 103]. This might be the reason for the usually low blood parasite counts in nestlings [106, 107]. Nevertheless, blood parasites were found in 20% of 13-day-old nestlings of the pied flycatcher (*Ficedula hypoleuca*) [104] and in 67% of 15-day old blue tits (*Cyanistes caeruleus*) [108]. Juveniles were sampled during their first year of life (i.e. post-fledging). For this reason, the age categories considered are ‘pre-fledging’, ‘juvenile’ and ‘adult’. Liver, heart and kidney samples were obtained from birds found dead in the vicinity of parrot nests, colonies or roosting places. For ethical reasons, we did not accept samples from hunted or lethally sampled birds for this study.

We collected blood samples *via* puncture of the cutaneous ulnar vein immediately after capture. In order to minimize stress, we sampled individuals only once and kept handling time to a minimum, usually below 5–10 min, before returning them to their nests. Like in previous studies, blood sampling had no detectable adverse effects [42, 93, 109, 110]. We stored blood samples on FTA classic cards (Whatman International Ltd., Maidstone, UK), in lysis buffer (100 mM Tris pH 8, 10 mM NaCl, 100 mM EDTA, 2% SDS), or in ethanol 70% (see details per species in Table 1). Liver, kidney and heart samples were sampled under sterile conditions (tools and cabinets) and preserved in 70% ethanol.

**Table 1:** Hemoparasites in 19 Psittaciformes from different habitats and climates of the Indo-Malayan, Australasian and Neotropical regions

	Locality (locality number)	Habitat^a^	Climate^b^	*n*	Age^c^	Tissue^d^	Hemoparasite presence and prevalence	Antiparasitic SM in diet?^e^	References
Cacatuidae									
Indo-Malayan									
Philippine cockatoo *Cacatua haematuropygia*	Rasa I., Palawan, Philippines (1)	M, MF	Am	16	pf	FTA	−	Yes	[140]
Psittacidae									
Australasian									
New Caledonian rainbow lorikeet *Trichoglossus haematodus deplanchii*	Ouégoa, New Caledonia (2)	Sa	Af	2	ad	FTA	−	No	[141, 142]
				1	pf	FTA	−		
	Parc des Grandes Fougères, New Caledonia (3)	Rf, Sa, PP	Af	5	ad	FTA	−		
	Motorpool, Nouméa, New Caledonia (4)	Su	Af	2	ad	FTA	−		
	Vallée des Colons, Nouméa, New Caledonia (5)	Su	Af	2	ad	FTA	−		
Forbes’ parakeet *Cyanoramphus forbesi*	Mangere I., Chatham Is., New Zealand (6)	CS	Cfb^f^	30	ad	FTA	−	No	[143–145]
Red-fronted parakeet *Cyanoramphus novaezelandiae*	Raoul I., New Zealand (7)	EP	Cfa^g^	34	ad	lys	Genus: *Plasmodium*; lineage: LIN4 (BELL02); identity 100%; prevalence 18%	No	[143, 146]
	Tiritiri Matangi I., New Zealand (8)	BF, Gr, NTP	Cfb	24	pf	lys	−		[111]
	Little Barrier I., New Zealand (9)	CK	Cfb	42	ad	lys	Genus: *Plasmodium*; lineage: LIN4 (BELL02); identity 100%; prevalence 5%; lineage: CN73; identity 99.7%; prevalence 5%		[102]
New Caledonian parakeet *Cyanoramphus saisseti*	Parc des Grandes Fougères, New Caledonia (3)	Rf, Sa, PP	Af	1	pf	FTA	−	No	[142]
Horned parakeet *Eunymphicus cornutus*	Parc des Grandes Fougères, New Caledonia (3)	Rf, Sa, PP	Af	1	ad	heart	−	No	[142]
	Parc Provincial de la Rivière Bleue, New Caledonia (10)	Rf, Ma	Af	1	pf	kidney	−		[142]
Ouvéa parakeet *Eunymphicus uvaeensis*	Gossana, Ouvéa, New Caledonia (11)	Rf, CP	Af	6	ad	FTA	−	No	[142]
				1	juv	FTA	−		
Neotropical									
Blue and yellow macaw *Ara ararauna*	Trinidad, Bolivia (12)	PFIS	Am	2	ad	FTA	−	Yes	[147]
	Sachojere, Bolivia (13)	PFIS	Am	1	pf	FTA	−		[147]
Blue-throated macaw *Ara glaucogularis*	Beni, Bolivia (14)	PFIS	Am	1	ad	lys	−	Yes	[147]
	Trinidad, Bolivia (12)	PFIS	Am	5	ad	FTA	−		[147]
				2	pf	FTA	−		
Blue-crowned conure *Thectocercus acuticaudatus*	Chaco, Argentina (15)	DXF	Cfa	1	ad	lys	−	Yes	[148]
				4	pf	lys	−		
White-eyed conure *Psittacara leucophtalmus*	Sachojere, Bolivia (13)	PFIS	Am	2	pf	FTA	−	No	[147]
Brown-throated conure *Eupsittula pertinax*	Isla Margarita, Venezuela (16)	CTS	Aw^f^	9	ad	FTA	−	Yes	[149]
Nanday conure *Aratinga nenday*	Principe Negro, Pantanal, Brazil (17)	Gr, Sa, SS, Ri	Aw	11	pf	FTA	−	Yes	[150]
Burrowing parrot *Cyanoliseus patagonus*	El Cóndor, Patagonia, Argentina (18)	Mo	BSk	32	ad	eth	−	Yes	[151]
				14	ad	liver	−		
	Comallo, Patagonia, Argentina (19)	PS	Csb	5	ad	liver	−		[152]
Austral parakeet *Enicognathus ferrugineus*	Navarino, Chile (20)	BFN	ET	2	ad	FTA	Genus: *Leucocytozoon*; prevalence 100%	No	[46]
	Bariloche, Patagonia, Argentina (21)	BFN	Csb	3	ad	FTA	Genus: *Leucocytozoon*; prevalence 100%		[153]
				1	pf	FTA	−		
				3	ad	liver	Genus: *Leucocytozoon*; prevalence 33%		
				1	pf	liver	−		
Blue-winged parrotlet *Forpus xanthopterygius*	Trinidad, Bolivia (12)	PFIS	Am	9	ad	FTA	−	Yes	[147]
Yellow-chevroned parakeet *Brotogeris chiriri*	Trinidad, Bolivia (12)	PFIS	Am	4	ad	FTA	−	Yes	[147]
Red-tailed Amazon *Amazona brasilensis*	Ilha Rasa, Guaraqueçaba, Brazil (22)	LAF, M	Cfa	29	pf	FTA	−	Yes	[154]
	Ilha das Gamelas, Guaraqueçaba, Brazil (23)	LAF, M	Cfa	4	pf	FTA	−		[154]
Blue-fronted Amazon *Amazona aestiva*	Jujuy, Argentina (24)	DXF	Cwa	6	ad	lys	−	Yes	[155]
	Chaco, Argentina (15)	DXF	Cfa	13	ad	lys	−		[148]
	Pantanal, Brasil (25)	Gr, Sa, SS, Ri	Aw	17	pf	FTA	−		[150]

^a^Habitats: *BF* remnants of broadleaf forest, *BFN* broadleaf forests dominated by *Nothofagus* spp., *CK* coastal and kauri (*Agathis australis*) forests, *CS* coastal scrub, *CP* coconut plantations, *CTS* cactus and thorn scrub, *DXF* deciduous xerophyte forest, *EP* endemic pohutukawa (*Metrosideros kermadecensis*) sub-tropical moist forest, *Gr* grassland with sparse trees, *LAF* lowland Atlantic forest, *M* mangrove, *Ma* maquis, *MF* monsoon forest, *Mo* Monte xerophyte forest, *NTP* native trees planted under a re-vegetation programme, *PFIS* palm dominated forest islands surrounded by regularly flooded savannah, *PP* pine plantations, *PS* Patagonian steppes, *Rf* rainforest, *Ri* riparian forest, *Sa* savannah, *SS* scrub savannahs, *Su* sub-urban

^b^The diversity of climates following [143, 156], except where indicated: Af, tropical rainforest; Am, tropical monsoon; Aw, tropical savannah; BSk, arid, steppe, cold; Cfa, temperate, without dry season, hot summer; Cfb, temperate, without dry season, warm summer; Csb, temperate, dry summer, warm summer; Cwa, temperate, dry winter, hot summer; ET, polar, tundra

^c^Age: age of individuals at the time of sampling; ad, adult; pf, pre-fledgling; juv, juvenile

^d^Tissue: tissue used to obtain DNA; FTA, blood in FTA cards; lys, blood in lysis buffer; eth, blood in ethanol 70%

^e^Food items known for their secondary metabolites (SM) with antimalarial, trypanocidal or general antiparasitic properties

^f^following [143] and WorldClim database (http://www.worldclim.org) [145], using diva-gis (http://www.diva-gis.org/)

^g^following [143] and data from the New Zealand National Climate Database (http://cliflo.niwa.co.nz)

*Abbreviation*: *n*, sample size

We only included samples obtained from wild Psittaciformes. We excluded samples of parrots and cockatoos that could have been in contact with (i) poultry; (ii) pets; (iii) zoos; (iv) aviculture facilities; (v) wildlife hospitals; (vi) rehabilitation facilities; (vii) reintroduction program facilities; or (viii) the staff of any of the previously mentioned facilities. One exception were the parakeets sampled at Tiritiri Matangi Island (New Zealand), which were descendants from birds translocated from captivity to the island *c.*30 years ago [111]. Co-workers who are veterinary practitioners and also work with captive birds, strictly refrained from contact with the above-mentioned facilities in the weeks before sampling. We considered these precautions mandatory, since parasites are common in captive birds, probably as an effect of captivity [86, 89–91]. We took special precautions to avoid both the contamination of samples and spreading of disease into wild populations during sampling.

Genomic DNA from samples stored in FTA cards was extracted according to Martínez et al. [112]. The DNA solution was purified using the commercial kit NZYGelpure (NZYTech, Lisbon, Portugal). By means of PCR, we amplified a part of the cytochrome *b* gene or the *18S* ribosomal RNA gene using previously published primers [113]. Sequences of the primers, size of the amplicons, and PCR conditions are shown in Table 2. PCR reactions consisted of 10 μl reaction volumes containing between 20 and 100 ng of template DNA, 0.25 μM of each primer and SYBR® Select Master Mix (Applied Biosystems, Foster City, CA, USA). The reactions were cycled using a StepOnePlus Real-Time PCR System (Applied Biosystems). The diagnosis was performed by visualizing the melting curve of the amplicons. After screening, positive samples were amplified again to obtain larger amplicons that facilitate the identification of haplotypes [113]. PCR reactions contained between 20 and 100 ng of the DNA template, Supreme NZYTaq 2× Green Master Mix (NZYTech) and 250 nM of each primer (Palu F/ Palu R). Using a Veriti thermal cycler (Applied Biosystems), reactions were run using the following conditions: 95 °C for 10 min (polymerase activation), 40 cycles at 95 °C for 30 s, annealing at 56 °C for 30 s, 72 °C for 30 s, and a final extension at 72 °C for 5 min. All amplicons were recovered from agarose gels and subjected to direct sequencing using an ABI 3730 XL automated sequencer (Applied Biosystems). DNA extraction and PCR set up were always performed in different laminar flow cabinets. We never detected amplicons in negative controls added in each PCR batch. A positive control for each pair of primers was routinely used. All analyses were carried out at the Departamento de Biomedicina y Biotecnología, Área de Parasitología, Facultad de Farmacia, Universidad de Alcalá, Alcalá de Henares, Spain, with the exception of the brown-throated conure (*Eupsittula pertinax*) samples. As we were not able to export these samples from Venezuela, some co-authors (AM, RR and VS) analysed the 9 samples of the brown-throated conure at the Instituto Venezolano de Investigaciones Científicas. In this case, *Haemoproteus*, *Plasmodium* and *Leucocytozoon* parasites were also screened by the nested PCR method but the partial amplification of the cytochrome *b* (here 471 bp) gene was carried out using different primers. For the first PCR round, we used the primer pair Haem NFI (5'-CAT ATA TTA AGA GAA ITA TGG AG-3') and Haem NR3 (5'-ATA GAA AGA TAA GAA ATA CCA TTC-3') [114]. We then used 2 μl of the first PCR reaction mixture as the template for a second-round PCR, in which Haem R2 (5'-GCA TTA TCT GGA TGT GAT AAT GGI-3') was paired with Haem F (5'-ATG GTG CTT TCG ATA TAT GCA TG-3'), and Haem R2L (5'-CAT TAT CTG GAT GAG ATA ATG GIG C-3') with Haem FL (5'-ATG GTG TTT TAG ATA CTT ACA TT-3') [114]. In order to detect possible positive samples that were not detected by the firsts primers set, we amplified an additional cytochrome *b* gene fragment also using a nested PCR [115]. The outer reaction was carried out with the primers DW2 (5'-TAA TGC CTA GAC GTA TTC CTG ATT ATC CAG-3') and DW4 (5'-TGT TTG CTT GGG AGC TGT AAT CAT AAT GTG-3’) [116]. We used a 2 μl aliquot of this product as a template for a nested reaction with primers DW1 (5’-TCA ACA ATG ACT TTA TTT GG-3') and DW6 (5'-GGG AGC TGT AAT CAT AAT GTG-3') [116]. Additionally, we carried out a nested PCR using 2 μl aliquot of the first reaction with the primers DW1 and HaemR (5'-CAT ATC CTA AAG GAT TAG AGC TAC CTT GTA A-3').

**Table 2:** Primers used for PCR detection of hemoparasites in wild Neotropical, Indo-Malayan and Australasian Psittaciformes

Gene	Primer name	Primer sequence 5’→3’	Size (bp)	Annealing	Parasite
Cytochrome *b*	Palu-Fq	CAAGGTAGCTCTAATCCTTTAGG	201	54 °C / 30 s	*Haemoproteus*; *Plasmodium*
	Palu-R	DGGAACAATATGTARAGGAGT			
Cytochrome *b*	L180	GAGAACTATGGAGTGGATGG	221	60 °C / 30 s	*Leucocytozoon*
	Leunew1-R	CCCAGAAACTCATTTGWCC			
*18S* rRNA	Try-F	GGAGAGGGAGCCTGAGAAATA	121	60 °C / 30 s	*Trypanosoma*
	Try-R	ATGCACTAGGCACCGTCG			
*18S* rRNA	NF110	GCTAATACATGCACCAAAGCTCC	119	60 °C / 30 s	microfilaria
	NR228	CAAGACCATGCGATCAGC			

We identified hemoparasite lineages using the Basic Local Alignment Search Tool (BLAST) in GenBank and the MalAvi databases [17]. We named new lineages using the five-letter species code to indicate the bird host, and submitted the results/sequences to GenBank and MalAvi databases.

The main food items consumed by the 19 Psittaciformes species that we sampled in this study is provided in Additional file 3: Table S2. Most of this information was gathered by the co-authors during ongoing research projects on the species considered, while some of the information originates from recent literature cited in Additional file 3: Table S2. Information on the secondary metabolites contained in the items consumed by the sampled parrots was obtained from the references also provided in Additional file 3: Table S2. The secondary metabolites were classified according to their main activity in (i) antimalarial, trypanocidal or general antiparasitic properties; (ii) anthelmintic; (iii) antimicrobial; and (iv) antioxidant (Additional file 3: Table S2). Detailed information on the habitats where we obtained our samples is provided in Table 1. Information on the climate associated with these habitats was obtained from the references provided in Table 1.

### Additional data from the literature

We also searched the literature for wild parrots previously investigated for hemoparasites, and additionally collected information on the corresponding diets, habitats and climates. For the literature search, we used the library of the Working Group Psittaciformes of the International Ornithologists’ Union. At the time of the literature search, this comprehensive library, which is updated monthly, comprised > 3600 papers (from 1817 to present). The literature in the library was reviewed manually by JFM in search for any previously published paper containing information on blood parasites affecting wild parrots. The search terms used to assist this search are listed in Additional file 1: Table S1.

We found 24 studies and reviews previously investigating blood parasites in wild parrots belonging to 52 species (covering 67 different populations; Additional file 1: Table S1). For these parrot species, we summarized in Additional file 1: Table S1 the following information: (i) number of individuals investigated (adults and nestlings); (ii) number of individuals infected, including the total number, and the subtotals discriminated in *Haemoproteus*, *Plasmodium*, *Leucocytozoon*, *Trypanosoma* and microfilaria; (iii) diets; (iv) habitats used; (v) climates; (vi) screening methods used for parasite detection and (vii) pertinent references. Yet, detailed information on specific food items consumed by a number of those parrot species is scarce (i.e. include a few plant items mentioned in the literature or vague observations like in e.g. *Aratinga euops* ‘they feed on fruits, seed, berries nuts and probably blossoms and leaf buds’, [75]) or even completely unknown (e.g. *Pionopsitta pulchra*, [75, 117]). For this reason, following González et al. [20], we classified the diets as (i) herbivorous; (ii) omnivorous-carrion, for species including carrion in their diets; (iii) omnivorous-insectivorous, for species including insects in their diets; (iv) ‘herbivorous?’-scarce information; or (v) unknown (Additional file 1: Table S1). The shortage of information on specific food items consumed by a number of these parrot species prevented us from being able to investigate the secondary metabolites that could be present in their diets (Additional file 1: Table S1), as we did for the parrot species sampled in this study (Additional file 3: Table S2).

### Comparative analyses

To facilitate comparisons, we included in Additional file 1: Table S1 the data corresponding to the 19 Psittaciformes species from the 25 localities that we investigated (marked ‘this study’ in the References column) and produced a combined dataset. Then, we transformed the infection information of the combined dataset into presence/absence data for each individual investigated (see combined dataset provided in Additional file 4), as 37% of previously published data on hemoparasites in wild parrots lacked information on prevalence. We excluded studies from statistical testing for which data were only partially available or uncertain (i.e. marked ‘NA’, ‘herbivorous?-scarce information’ or ‘unknown’ in Additional file 1: Table S1). We also excluded studies from statistical testing for which only data on nestlings existed. This was done to avoid cases in which infection could have been not detected because the nestlings were too young at the time of sampling thus not giving the parasites time to develop. Although we were certain that this was not the case for the nestlings sampled in our study, and in order to be conservative, we likewise excluded our data on nestlings from statistical testing. Given the large number of habitat and climate categories in the combined dataset, we transformed the data into binomial categories to allow adequate statistical comparisons. Habitats were classified as forest or non-forest, climates as tropical or non-tropical, diets as herbivorous or omnivorous, and the screening methods as PCR-based or not (Additional file 4). We assessed the combined dataset (*N* = 520) for the effects of diet, habitat, climate and screening method (as factors) on the presence of parasites in the studied individuals using Generalized Linear Mixed-Effects Models with binomial error distribution and model selection based on Akaike information criterion (AIC) in the R programming language (script and full dataset provided in Additional file 4; packages *MuMIn* and *lme4*) [118]. To account for inherent variation among species, we also included the species to which each individual subject belonged as a random intercept in the Generalized Linear Mixed-Effects Models (script provided in Additional file 4). In the dredge function in *MuMIn*, all possible candidate models (i.e. subsets of the global model) were tested using each unique linear combination of covariates. The best models are then selected based on ΔAIC scores less than or equal to two. In order to evaluate the predictive power of our diet models and its balance between sensitivity and specificity, we ran a 10-fold cross validation, fitting the model to training sets and predicting for with-held test sets (see script provided in Additional file 4). The 10-fold cross validation provides a mean area under the receiver operating characteristic curve (mean AUC). Odds ratios were calculated to provide a measure of how the probability of infection is predicted to change, for instance when a species has an omnivorous diet compared to a species without an omnivorous diet (for calculation see Additional file 4). Additionally, to allow an adequate evaluation of our results, we simulated the probability that the parasites will actually be detected given the sample size and an expected true prevalence of 0.08213 based on prevalence data previously reported in wild Psittaciformes (Additional file 1: Table S1). The simulation script is provided in Additional file 4 and the probabilities of detection for all species and sample sizes are provided in Additional file 1: Table S1.

## Results

Hemoparasites were present in adult birds of only two of the 19 species sampled for this study (Table 1). In the red-fronted parakeet, hemoparasites were detected in samples from Raoul Island and Little Barrier Island, but not on Tiritiri Matangi Island (New Zealand; Additional file 2: Figure S4; Table 1). The red-fronted parakeets from Raoul Island were infected with hemoparasites from the genus *Plasmodium* corresponding to the haplotype LIN4 (BELL02, new lineage name in MalAvi database; identity 100%; prevalence 18%; GenBank accession number MH238461). The birds from Little Barrier Island were infected with two *Plasmodium* haplotypes, one that corresponded to LIN4 [102] (identity 100%, prevalence 5%), and another one which differed in a single nucleotide, considering a 312 bp length sequence, from the haplotype GRW06 [119] (thereafter haplotype CN73, GenBank accession number MH238460, identity 99.7%, prevalence 5%).

All adult austral parakeets (*Enicognathus ferrugineus*), both from Navarino Island and Bariloche, Patagonia (separated by more than 1500 km; populations 20 and 21 in Additional file 2: Figure S5), were infected with hemoparasites from the genus *Leucocytozoon* belonging to an un-described haplotype (Merino et al. description in prep.). This new *Leucocytozoon* haplotype differs 3 to 4% in its sequence with respect to several haplotypes previously described in birds from the genus *Atrix* (e.g. GHOW93-00-55, NSPOWORRO15, BAOW5909, SPOW7; Merino et al. unpublished data). Compared to the blood samples, only a third of the austral parakeet liver samples contained the same *Leucocytozoon* haplotype (Table 1).

All other 17 species of Psittaciformes sampled in this study, covering a wide range of habitats and climates in the Indo-Malayan, Australasian and Neotropical zoogeographical regions, were not infected by any of the tested hemoparasites (*Haemoproteus*, *Plasmodium*, *Leucocytozoon*, *Trypanosoma* and microfilariae; Table 1). No pre-fledging nestling or juvenile was infected, including those from the red-fronted parakeet and austral parakeet.

The main food items consumed by the 19 parrot species that we sampled are provided in Additional file 3: Table S2. Most parrot diets were herbivorous, except for the two species in which blood parasites were detected: the red-fronted parakeet and the austral parakeet which also consume items of animal origin (Additional file 3: Table S2). In addition to fruits, flowers and unripe seeds, red-fronted parakeets consume invertebrates and marine molluscs and occasionally scavenged animal carrion, including birds (omnivorous-carrion diet). Austral parakeets include in their diet larvae of Homoptera, Lepidoptera and Hymenoptera (*Aditrochus fagicolus*) (omnivorous-insectivorous diet; Additional file 3: Table S2). Thus, in our samples, only omnivorous species were infected with hemoparasites.

Fifteen of the 19 parrot species that we sampled (79%) regularly consume food items that include secondary metabolites known for their antiparasitic activity, including antimalarials, antifungals, leishmanicidals, trypanocidals, anthelmintics, insecticides and mosquitocidals (Additional file 3: Table S2). Of the 15 species, those that consume food with antimalarial or general antiparasitic properties were free from *Haemoproteus*, *Plasmodium*, *Leucocytozoon*, *Trypanosoma* and microfilariae: Philippine cockatoo (*Cacatua haematuropygia*), blue and yellow macaw (*Ara ararauna*), blue-throated macaw (*Ara glaucogularis*), blue-crowned conure (*Thectocercus acuticaudatus*), brown-throated conure (*Eupsittula pertinax*), nanday conure (*Aratinga nenday*), burrowing parrot (*Cyanoliseus patagonus*), blue-winged parrotlet (*Forpus xanthopterygius*), yellow-chevroned parakeet (*Brotogeris chiriri*), red-tailed Amazon (*Amazona brasilensis*), and blue-fronted Amazon (*Amazona aestiva*) (Table 1, and Additional file 3: Table S2). None of the species found infected (red-fronted parakeets, austral parakeets) consumed any food item with antimalarial or general antiparasitic properties (Table 1, and Additional file 3: Table S2). However, red-fronted parakeets consumed a food item known for the presence of secondary metabolites with anthelmintic activity (Additional file 3: Table S2). In addition, all 19 studied parrot species consumed items that contained secondary metabolites known for their antimicrobial activity, and 17 species (89%) involved items with antioxidant properties (Additional file 3: Table S2). To a lesser extent, some parrots consumed food items containing secondary metabolites recognized for their e.g. antiinflammatory, anticarcinogenic, analgesic, expectorant, or antipyretic effects (Additional file 3: Table S2).

When the data corresponding to the Psittaciformes sampled in this study were combined with the data from 24 studies previously investigating blood parasites in wild parrots (Additional file 4), we found that 7 of 58 herbivorous wild parrots (12%) were reported to be infected with *Haemoproteus*, *Plasmodium* or microfiliaria. In contrast, 7 of 10 omnivorous wild parrots (70%) were reported to be infected with *Leucocytozoon*, *Haemoproteus* or *Plasmodium* (Additional file 1: Table S1). Model selection revealed that the four best models included diet, whereas the second best included diet and habitat, the third best included diet and screening method, and the fourth best included diet and climate (Additional file 4). The model-averaged coefficients (7.5, SE = 3.7; *P* = 0.04) and the odds ratio (OR = 1882.6) suggested a strong positive relationship between omnivorous diets and hemoparasite infestation), but a weaker relationship with forest habitat (OR = 0.5), non-tropical climate (OR = 0.8), and non-PCR-based screening method (OR = 0.8). The 10-fold cross-validation showed for our diet model a high predictive power and a good balance between sensitivity and specificity (mean AUC = 0.9).

## Discussion

Two clear patterns emerged from our results. First, our results appear to support the hypothesis that parrots engaging into self-medication are free from hemoparasites. All the studied parrots that regularly consume at least one food item known for its secondary metabolites with antimalarial, trypanocidal or general antiparasitic properties were indeed free from *Haemoproteus*, *Plasmodium*, *Leucocytozoon*, *Trypanosoma* and microfilariae (Table 1). Moreover, no food items with antimalarial or even general antiparasitic properties were consumed by red-fronted parakeets, which were infected with *Plasmodium* haplotypes, or by austral parakeets, found infected with *Leucocytozoon*. These results suggest that self-medication in parrots may be more widespread than previously thought. Nevertheless, some species that do not regularly incorporate antiparasitic metabolites in their diets were also free from blood parasites [e.g. New Caledonian rainbow lorikeet (*Trichoglossus haematodus deplanchii*), Forbes’ parakeet (*Cyanoramphus forbesi)*, New Caledonian parakeet (*Cyanoramphus saisseti*), horned parakeet (*Eunymphicus cornutus*), Ouvéa parakeet (*Eunymphicus uvaeensis*) and white-eyed conure (*Psittacara leucophtalmus*)]. In the case of the white-eyed conure, available data on their diet are limited (Additional file 3: Table S2) and thus, we may have missed food items that could provide this species with antiparasitic protection. In the case of the Forbes’ parakeet from Mangere Island, the lack of necessary vectors previously reported [120] could explain the results. Additionally, small sample sizes could have played a role in at least three of these species (Table 1). Possible sampling bias should be considered when interpreting our findings and should steer future work to assess how these patterns hold following additional surveys, particularly among poorly sampled parrot species such as the New Caledonian parakeet, horned parakeet, Ouvéa parakeet and orange-fronted parakeet (*Aratinga canicularis*). Nevertheless, results from previous studies lend support to our primary conclusions by demonstrating that some animals deliberately consume food items with prophylactic or therapeutic activity against pathogens or parasites [59–68]. These include a wide range of species such as wood ants (*Formica paralugubris*) engaging in social prophylaxis, therapeutic self-medication in the woolly bear caterpillar (*Grammia incorrupta*), or prophylactic self-medication in the Ethiopian baboon (*Papio anubis* [61]). In birds, great bustards increase their mating success and thus fitness by consuming blister beetles (Meloidae), which are known to contain secondary metabolites with antimicrobial and pathogen-limiting activity ([62] but see [121]). Some bird species have been reported to be toxic, including the European quail (*Coturnix coturnix*), several New Guinea species from the genus *Pitohui*, the North-American ruffed grouse (*Bonasa umbellus*), the spur-winged goose (*Plectropterus gambensis*) from Benin, and some Australian bronzewings from the genus *Phaps* [122]. The toxicity is produced by batrachotoxin, cantharidin, andromedotoxin and alkaloids contained in the skin and feathers, which have been suggested to protect the birds against predators and parasites [122]. Also, parrots have been shown to use plants for prophylactic reasons. Previous studies on monk parakeets and red-fronted parakeets described the use of plants known for their insecticidal activity, which protect their nests from parasites [94, 95, 123, 124]. Furthermore, red-fronted parakeets chew leaves of these plants, mix the chopped material with preen oil and spread the mixture on their feathers to repel insects [95]. Thus, our results add further evidence in favour of prophylactic anti-parasite self-medication as a wider than previously thought behaviour in both vertebrates and invertebrates, and increase our understanding of why certain food items are taken regardless being poisonous or with little nutritional value [61–63, 97–99]. Even more, anti-parasite self-medication can be related to human food consumption and health. For instance, the increase in disease in honeybees (*Apis mellifera*) as a consequence of agricultural practices that interfere with the ability of bees to self-medicate [61]. Research on animal self-medication has the potential to trigger the discovery of new secondary metabolites contained in the food items consumed by animals. Some of these secondary metabolites may have pharmacological properties and therefore, could contribute to human health care. [63]. However, it is important to mention that to fully test prophylactic anti-parasite self-medication in parrots, further work, particularly experimental research, should be conducted. It would also be necessary to investigate species living in the same or close regions/habitats to those infected in order to test if they are free of parasites when ingesting antiparasitic substances, i.e. that they are exposed to the same vectors but remain uninfected. Alternatively, infection by blood parasites in these species could be lethal, making the discovery of infected birds unlikely.

The second interesting pattern that we observed is that the two infected parrot species (red-fronted and austral parakeets) regularly consume omnivorous diets. This contrasts with the non-infected species, which are all herbivorous (Additional files 1: Table S1 and Additional file 3: Table S2). Furthermore, when we combined our data with data from previous studies, the strong positive relationship between omnivorous diets and hemoparasite infestation was confirmed. Our results are in fully agreement with a previous wide-ranging study including 246 species of wild Neotropical birds, which found that species with omnivorous diets had higher prevalence of *Plasmodium*, whereas insectivores had higher prevalence of microfilariae than birds with a different feeding behaviour [20]. More recently, Naqvi et al. [125] found that the prevalences of *Haemoproteus*, *Plasmodium*, and *Leucocytozoon* in chickens were higher in free-ranging individuals that also feed on carrion. Therefore, the consumption of carrion and its associated scavenging behaviour by red-fronted parakeets or the consumption of insect larvae by austral parakeets could increase their exposure to pathogen transmitting vectors. However, our review of previous studies on parrots did not show a full association between the diet consumed and the presence of hemoparasites: 70% of omnivorous wild parrots were reported to be infected, while only 12% of herbivorous were also reported as infected (Additional file 1: Table S1). This could mean that at least a second factor is involved, probably including the intake of antiparasitic secondary metabolites or differences in the habitats or climates used, e.g. [20], as our other results suggested. Sampling effort bias could also be a reason for the lack of a full association between the diet consumed and the presence of hemoparasites, reinforcing the need of more studies on the foraging ecology of parrots. Nevertheless, several studies on parrots found that the importance of food items of animal origin has been largely overlooked and that probably more parrot species than currently known have omnivorous diets at least during some parts of the year [98, 126–137]. Further studies investigating hemoparasites in other parrot species considering their diet might provide the necessary dataset to fully test this hypothesis. Lastly, an omnivorous diet could reduce the amount of plant material consumed by a given animal, reducing the intake of secondary metabolites with antiparasitic, antimicrobial, antiinflammatory, anticarcinogenic, analgesic, expectorant or antipyretic activities, and thus have a negative impact on health.

Our comparative analyses including previously published studies also show that parrots living in forests have a higher probability of being infected with hemoparasites than those living in open (non-forested) habitats. Individuals from the two species infected in our samples, the red-fronted parakeet and the austral parakeet belonged to wild populations that inhabit forests (Table 1). This result is in line with previous research showing that avian species breeding in forested habitats have higher hemoparasite prevalences, as revealed for instance in Spanish raptors [70]. Similarly, as a recent review [47] describes, a lower avian malaria prevalence in the olive sunbird (*Cyanomitra olivacea*) was associated with deforestation in Ghana while the prevalence was higher in the intact forested areas of Cameron. It has been shown that habitats like forests influence the parasite prevalence in the insect vectors, or even the presence of the vectors, which in turn can influence the transmission to avian hosts [47–50, 54, 70]. Moreover, vector abundance appears to be lower in open habitats and areas that are under anthropogenic impact [70]. In contrast, potential dipteran vectors abound in the sector of northern Patagonia, where the austral parakeet population was investigated, and in the islands of New Zealand, where the red-fronted parakeet was investigated [138, 139]. Also, previous research on the hemoparasites of southern Chile showed that *Haemoproteus*, *Plasmodium* and *Leucocytozoon* were present in up to 13% of the sampled birds from the *Nothofagus* forest in Navarino [46], where our second austral parakeet population was sampled, thus suggesting that the necessary vectors were present.

## Conclusions

The consumption of food items known for their secondary metabolites with antimalarial, trypanocidal or general antiparasitic properties appeared to be linked to the absence of hemoparasites. Furthermore, our results suggest that prophylactic anti-parasite self-medication could be a widespread behaviour in animals. On the other hand, a relationship between the consumption of omnivorous diets and hemoparasite infestation also appeared to be a more frequent pattern than previously thought. Consequently, both factors acting together may be a valid explanation for the absence of hemoparasites reported in a number of vertebrate species. This result has the potential to trigger new lines of research in search of the mechanism driving hemoparasite infections, such as the investigation of other species living sympatrically with those infected to test if they are free of parasites when ingesting antiparasitic secondary metabolites. This study also highlights a considerable deficit in necessary data on food items consumed. More of such data will allow more definite links between hemoparasite infections and diets.

## Additional files

Additional file 1: **Table S1.** Hemoparasites in wild Psittaciformes. Malaria parasites (*Plasmodium*), related intracellular haemosporidians (*Haemoproteus* and *Leucocytozoon*), the unicellular parasitic flagellate protozoans (*Trypanosoma*), and microfilaria reported in wild populations of Psittaciformes. The probability of detection for adults is based on a simulation (see Additional file 4) of the probability that the parasites will actually be detected given the sample size and an expected true prevalence based on the prevalences observed in wild Psittaciformes. The habitat and climate classification follow the references in Table 1. (XLSX 34 kb)

Additional file 2: **Figure S1.** Locations of the sampled population at Rasa I., Palawan, Philippines, in the Indo-Malayan zoogeographical region. **Figure S2.** Locations of the sampled populations in New Caledonia, Australasian zoogeographical region. **Figure S3.** Locations of the sampled population in the Chatham Is., Australasian zoogeographical region. **Figure S4.** Locations of the sampled populations in New Zealand, Australasian zoogeographical region. **Figure S5.** Locations of the sampled populations in the Neotropical zoogeographical region. (PDF 1271 kb)

Additional file 3: **Table S2.** Main food items consumed by the Psittaciformes species in the localities where the blood parasite sampling was carried out. Details on the species, main food items and parts consumed are provided. The presence of secondary metabolites with antimalarial/general antiparasitic plant secondary metabolites, anthelmintic, antimicrobial, and antioxidant properties is indicated. Source references are provided. (XLSX 29 kb)

Additional file 4: Scripts and combined dataset to analyse the presence of hemoparasites in Psittaciformes. Analyses and the combined dataset for the effects of diet, habitat, climate, screening method (as factors) and species (as a random variable) on the presence of parasites in the studied individuals using a binomial General Lineal Mixed-Effects Model and model averaging based on Akaike information criterion (AIC) with R. Scripts for the 10-fold cross validation and the calculations of parasite detection probability are also provided. (TXT 34 kb)

## Abbreviations

AIC: Akaike information criterion
AUC: Area under the curve
BLAST: Basic local alignment search tool
NA: Missing data
OR: Odds ratios
PCR: Polymerase chain reaction
pf: Pre-fledging
