# Supplementary material for: Can the intake of antiparasitic secondary metabolites explain the low prevalence of hemoparasites among wild Psittaciformes?
Source: Parasit Vectors. 2018 Jun 19;11:357. doi: 10.1186/s13071-018-2940-3 (PMC6008929; doi:10.1186/s13071-018-2940-3)

Additional file 2

Can the intake of anti-parasitic secondary metabolites explain the low prevalence of hemoparasites among wild Psittaciformes?

Juan F. Masello, Javier Martínez, Luciano Calderón, Michael Wink, Petra Quillfeldt, Virginia Sanz, Jörn Theuerkauf, Luis Ortiz-Catedral, Igor Berkunsky, Dianne Brunton, José A. Díaz-Luque, Mark E. Hauber, Valeria Ojeda, Antoine Barnaud, Laura Casalins, Bethany Jackson, Alfredo Mijares, Romel Rosales, Gláucia Seixas, Patricia Serafini, Adriana Silva-Iturriza, Elenise Sipinski, Rodrigo A. Vásquez, Peter Widmann, Indira Widmann, Santiago Merino

Figure S1. Locations of the sampled population at Rasa I., Palawan, Philippines, in the Indo-Malayan zoogeographical region. Localities are numbered as in Table 1

Figure S2. Locations of the sampled populations in New Caledonia, Australasian zoogeographical region. Localities are numbered as in Table 1

Figure S3. Locations of the sampled population in the Chatham Is., Australasian zoogeographical region. Localities are numbered as in Table 1

Figure S4. Locations of the sampled populations in New Zealand, Australasian zoogeographical region. Localities are numbered as in Table 1

Figure S5. Locations of the sampled populations in the Neotropical zoogeographical region. Localities are numbered as in Table 1

Fig. S1. Indo-Malayan region

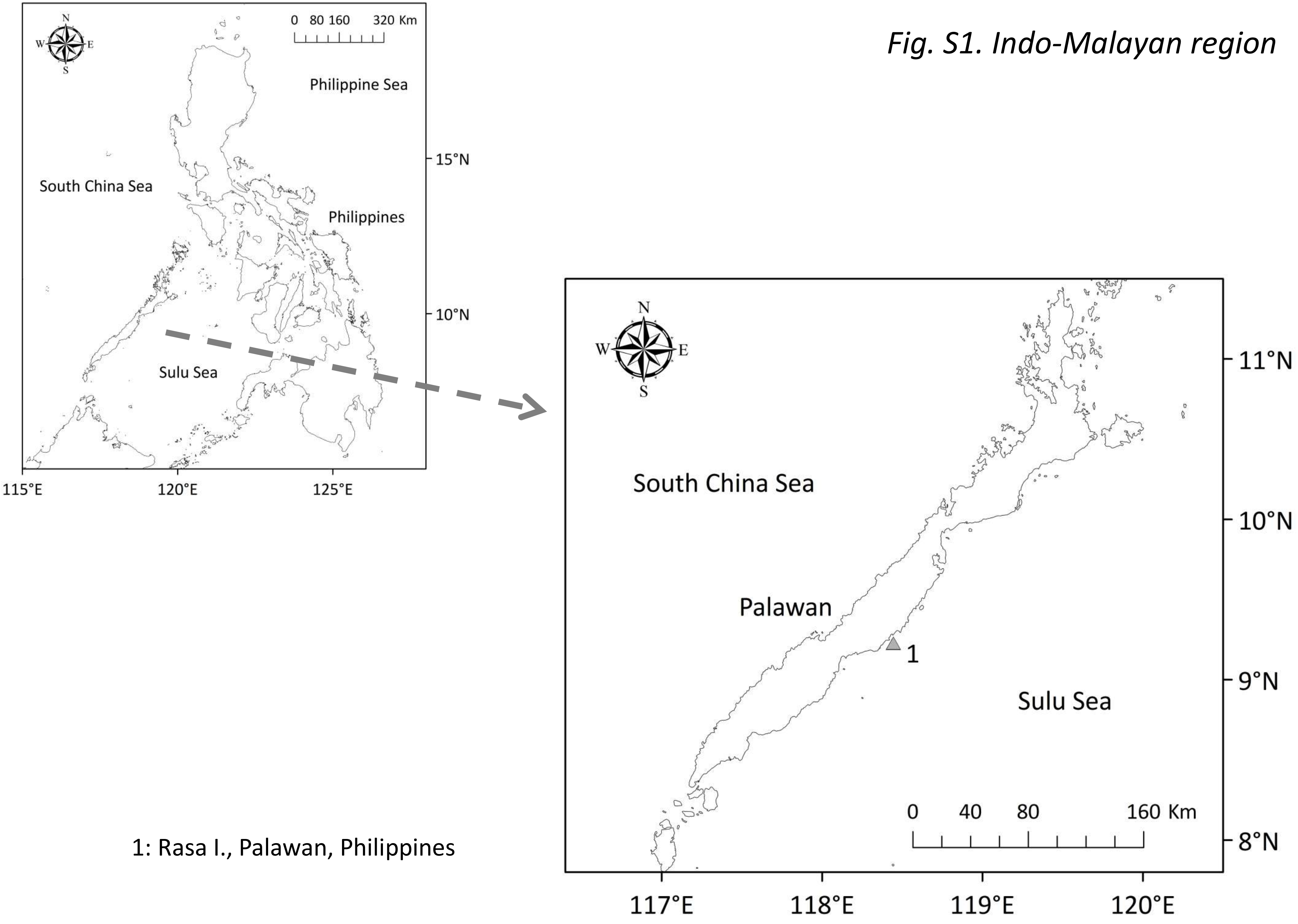

1: Rasa I., Palawan, Philippines

Fig. S2. Australasian region: New Caledonia

2: Ouegoa

3: Parc des Grandes Fougères

4: Motorpool, Nouméa

5: Vallée des Colons, Nouméa

10: Parc Provincial de la Rivière Bleue

11: Gossana, Ouvéa

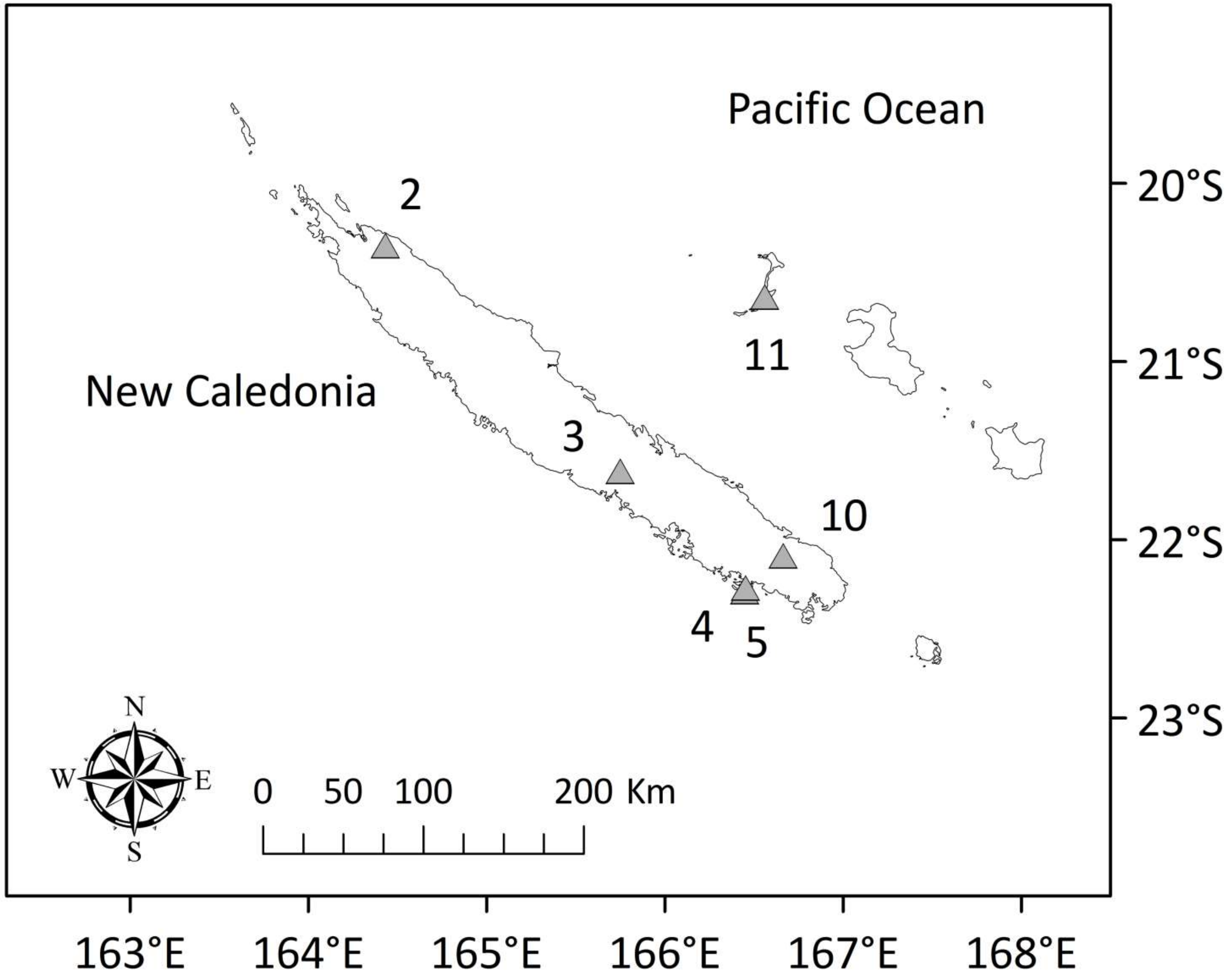

Fig. S3. Australasian region: Chatham Is., New Zealand

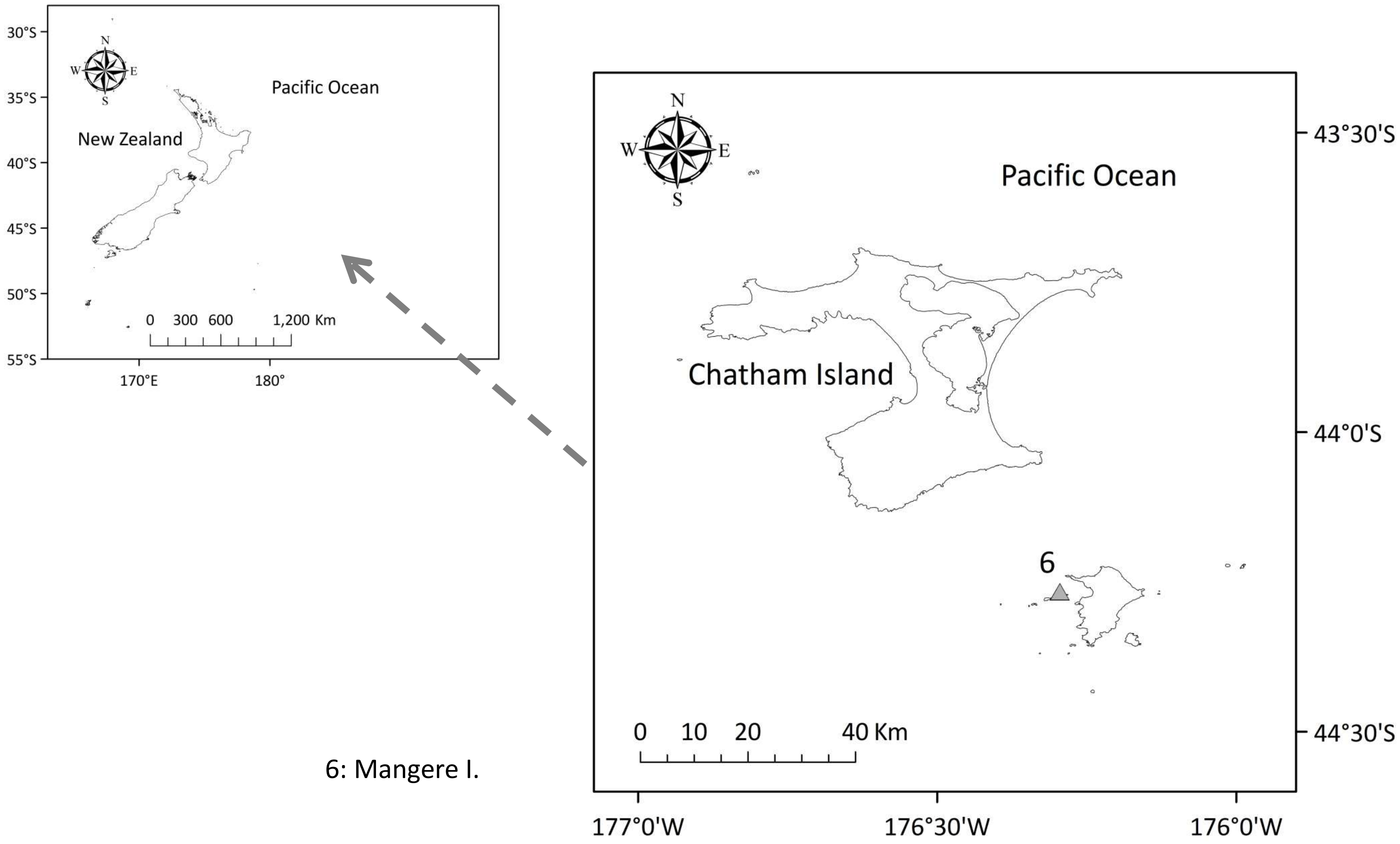

6: Mangere I.

Fig. S4. Australasian region: New Zealand

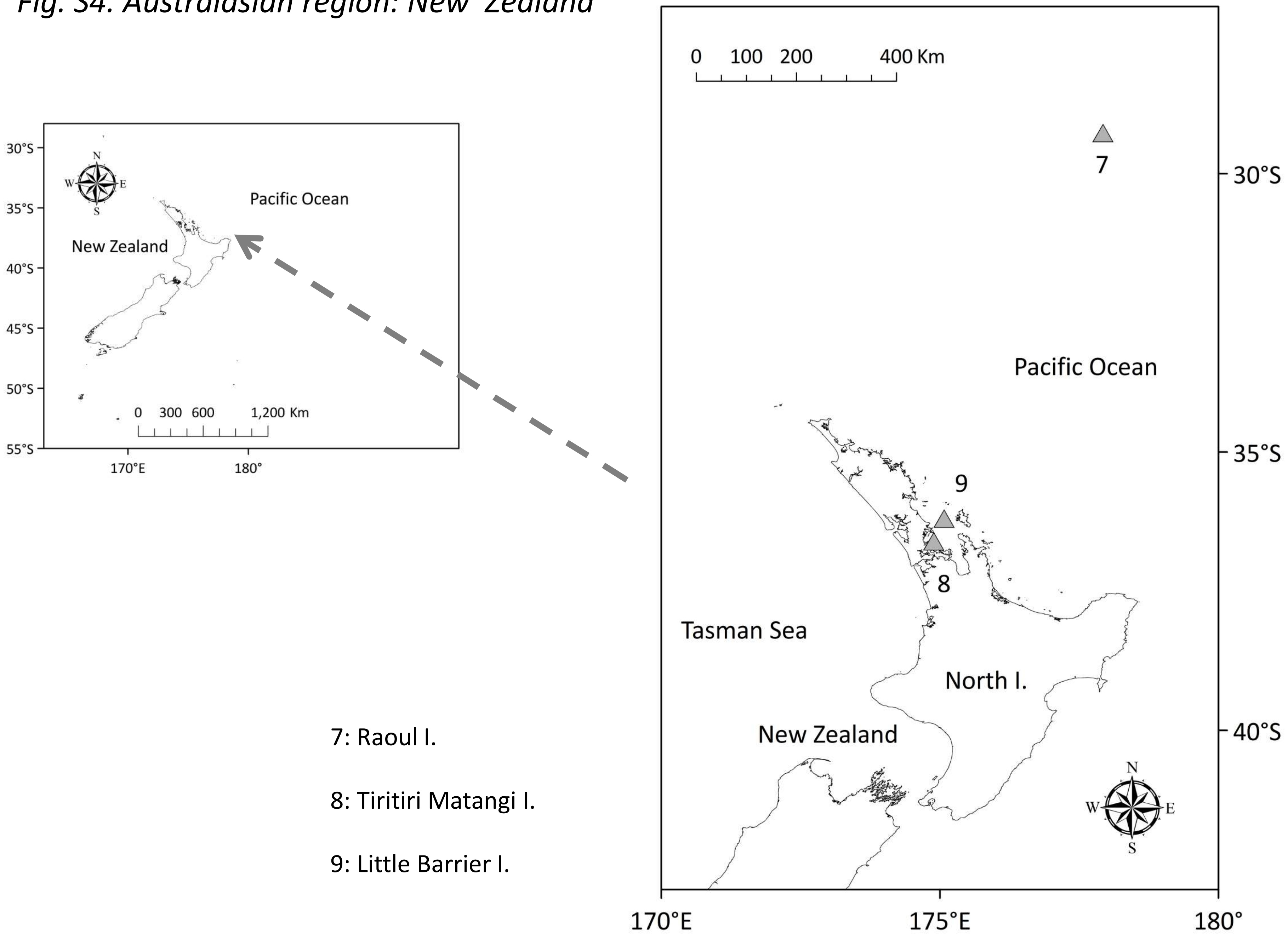

Fig. S5. Neotropical region

12: Trinidad, Bolivia

13: Sachojere, Bolivia

14: Beni, Bolivia

15: Chaco, Argentina

16: Isla Margarita, Venezuela

17: Principe Negro, Pantanal, Brazil

18: El Cóndor, Patagonia, Argentina

19: Comallo, Patagonia, Argentina

20: Isla Navarino, Chile

21: Bariloche, Patagonia, Argentina

22: Ilha Rasa, Guaraqueçaba, Brazil

23: Ilha das Gamelas, Guaraqueçaba, Brazil

24: Jujuy, Argentina

25: Pantanal, Brasil

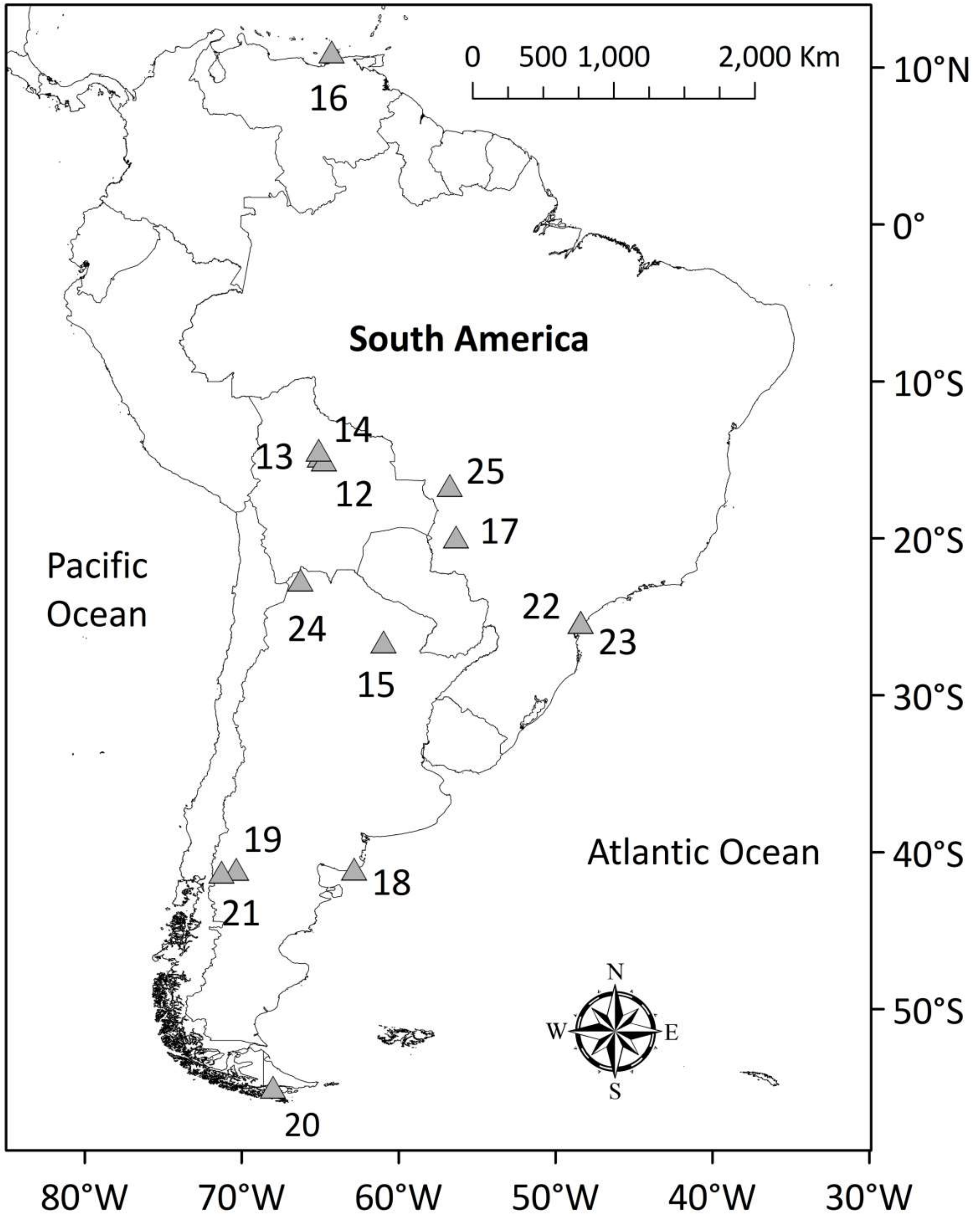

Supplement: Supplementary file 2 — Figure S1. Locations of the sampled population at Rasa I., Palawan, Philippines, in the Indo-Malayan zoogeographical region. Figure S2. Locations of the sampled populations in New Caledonia, Australasian zoogeographical region. Figure S3. Locations of the sampled population in the Chatham Is., Australasian zoogeographical region. Figure S4. Locations of the sampled populations in New Zealand, Australasian zoogeographical region. Figure S5. Locations of the sampled populations in the Neotropical zoogeographical region. (PDF 1271 kb) [file 13071_2018_2940_MOESM2_ESM.pdf]
